# Secondary headache due to aspergillus sellar abscess simulating a pituitary neoplasm: case report and review of literature

**DOI:** 10.1186/s40064-015-1343-6

**Published:** 2015-09-24

**Authors:** Wenyao Hong, Yuqing Liu, Mingwu Chen, Kun Lin, Zhengjian Liao, Shengyue Huang

**Affiliations:** Department of Neurosurgery, Fujian Provincial Hospital, Fujian Medical University, 134 Dongjie Road, Gulou District, Fuzhou, 350001 China

**Keywords:** Aspergillus, Fungal, Sellar abscess, Pituitary neoplasm

## Abstract

Fungal sellar abscess is rare. A 42-year-old man was admitted with 2-month headache characterized by right peri-orbital pain. An intrasellar mass was found to be simulated a pituitary neoplasm after magnetic resonance imaging examination, and operated on via an endoscopic trans-sphenoidal approach. Milk-like pus and a mass of ash black mixed and necrotic material were found and removed. Histopathology revealed numerous aspergillus hyphae. Itraconazole was given on a dosage of 200 mg twice a day orally for 6 weeks. No recurrence was observed during follow-up. Complete surgical resection through endoscopic trans-sphenoidal approach combined with systemic anti-fungal therapy, should be considered as the optimal treatment.

## Background

Fungal sellar abscess is rare and only 18 cases have been reported. In this report, we state a case of invasive aspergillosis involving the sellar area, which was revealed by clinical features suggesting a pituitary neoplasm.

## Case report

**Fig. 1:**
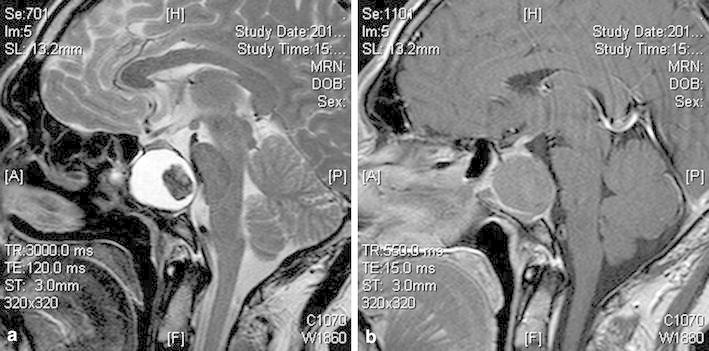
**a** On T2 weighted image, the central part of the mass was hypo-intense, the peripheral part was hyper-intense; **b** contrast enhanced T1-weighted MRI showed the mass was of slight rim enhancement. The sella turcica was enlarged and the sphenoidal sinus was greatly diminished

A 42-year-old man was admitted with headache and diplopia of 2 days duration in December 2014. The feature of headache was persistent and electric shock-like peri-orbital pain on the right side, without nausea and vomit. He was engaged in fungus mushroom cultivation for 3 years. His medical history was insignificant. Neurological examination showed the right abducens nerve palsy. T2-weighted MRI revealed an heterogenous intrasellar mass (Fig. 1a). Contrast enhanced T1-weighted MRI showed the mass was of slight rim enhancement (Fig. 1b). Levels of endocrinological workup were all within normal limits. Before operation, headache could not be controlled by the drug of mannitol and NSAIDs except oxcarbazepin.

**Fig. 2:**
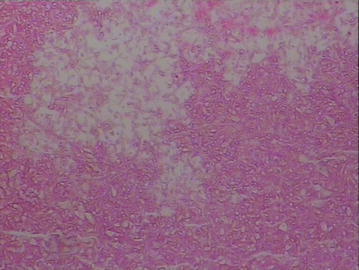
(Hematoxylin and eosin ×100) Photomicrograph of the intra sellar mass showing fungal organisms with septate hyphae and spores, which was consistent with aspergillus

With a preoperative diagnosis of pituitary neoplasm, endoscopic trans-sphenoidal surgery was planned. After the diminished sphenoidal sinus was entered, some milk-like pus and thickened mucosa were excised. When the paper-thin sellar floor bone and dura were opened, more milk-like pus and a mass of ash black mixed and necrotic material were found and removed. The pituitary gland and the diaphragma sellae were found to be intact after the removal of the lesion. The specimens were negative for aerobic and anaerobic bacterial cultures. Histopathological examination of the necrotic material consisted of numerous hyphae, according with characteristic of aspergillus species (Fig. 2). Plasma (1 → 3)-beta-d glucan determination was done and showed a elevated level (203.5 Pg/mL, normal, 0–100.5 Pg/mL). Itraconazole was given on a dosage of 200 mg twice a day orally for 6 weeks.

Postoperatively he get a transient diabetes insipidus for 5 days. He had relief from headache and improvement in function of the right abducens nerve. No recurrence was observed on contrast enhanced MRI scans during 6-month follow-up.

## Discussion

The major pathogen of fungal sellar abscess is aspergillus species. Aspergillus are ubiquitous fungi found in soil and organic materials and can establish saprophytic growth within the respiratory tract after inhalation. The organism can become pathogenic under anaerobic conditions. In our case, the patient had been engaging in fungus mushroom cultivation for 3 years, which may be a cause of sellar aspergillosis, since there was no significant medical history.

Preoperative diagnosis of fungal sellar abscess is difficult (Larrañaga et al. 1989). The presenting symptoms are usually identical to the symptoms in patients with pituitary neoplasm. Headache is the most commonly reported symptom. It is related with tumor extension and intrasellar pressure, sellar structures, patient predisposition, familial history, and functional disturbance within the hypothalamo-pituitary axis (Gondim et al. 2009). In our case, headache was characterized by electric shock-like peri-orbital pain and sensitive to oxcarbazepin, may be caused by invasion to the cavernous sinus and compression on ophthalmic branch of trigeminal nerve. Additionally, there are several complications such as diplopia (Ahmadzai et al. 2013; Fuchs et al. 1985; Iplikcioglu et al. 2004; Li et al. 2008; Petrick et al. 2003; Scanarini et al. 1991), visual deterioration (Ahmadzai et al. 2013; Endo et al. 2001; Goldhammer et al. 1974; Kalayci and Cevik 2014; Lee et al. 2000; Liu et al. 2013; Pinzer et al. 2006; Scanarini et al. 1991), and endocrinological abnormality (Li et al. 2008). None was feverish before admitted to hospital. However, MRI examination may help preoperative diagnosis of sellar abscess. T2-weighted MRI image showing a regular hypo-intense zone into the hyper-intense process, may correspond to iron accumulation and be specific to aspergillus infection (Boutarbouch et al. 2009; Kurita et al. 1995; Li et al. 2008; Petrick et al. 2003). Contrast enhanced T1-weighted MRI showed the mass was of slight rim enhancement (Li et al. 2008; Petrick et al. 2003; Vijayvargiya et al. 2013), which may suggest a chronic inflammation.

The most effective treatment of fungal sellar abscess is endoscopic trans-sphenoidal surgery. Craniotomy may increase the risk of subarachnoid spread of infection which may be lethal (Vijayvargiya et al. 2013). In these reported cases, only 2 (Endo et al. 2001; Goldhammer et al. 1974) patients underwent frontal craniotomy, who were separately died from occlusion of ICA and fulminant fungal meningoencephalitis. Trans-sphenoidal approach minimizes the likelihood of CSF contamination. Endoscopy can help the sellar abscess exposed and cleared completely. However, Major efforts must be taken to carefully keep the diaphragma sellae intact to avoid from contamination of CSF.

The controversy is that patients with fungal sellar abscess should be treated with anti-fungal drug when the diagnosis was ascertained. Some reports consider postoperative systemic anti-fungal therapy as superfluous in case of total macroscopic extirpation of an invasive aspergillosis without subdural involvement (Boutarbouch et al. 2009; Kalayci and Cevik 2014; Pinzer et al. 2006). However more authors approve of a combination of surgery and postoperative systemic anti-fungal therapy. In our case, level of plasma (1 → 3)-beta-d glucan was elevated postoperatively, which indicated an invasive fungal infection. Hence, anti-fungal therapy was followed by immediately after the histopathological diagnosis. Amphotericin B, voriconazole, itraconazole, were all effective for aspergillus. Amphotericin B treatment is associated with significant toxicity and prolonged hospital stay (Ramos-Gabatin and Jordan 1981). Although voriconazole can be safe and effective anti-fungal agent (Li et al. 2008), itraconazole was chose based on our patient’s poor economic station. One month later, the level of plasma (1 → 3)-beta-d glucan was declined and within the normal limit (83.6 Pg/ml).

## Conclusion

Long-term cultivation of fungus mushroom may be a cause of sellar aspergillosis. If people who long-time working in damp and dark environment, is attacked by acute and persistent headache, diagnosis of fungal sellar abscess should be taken into consideration. Although the diagnosis is difficult, MRI showing a hypo-intense zone into the hyper-intense process and rim enhancement, may help to preoperative diagnosis. Complete surgical resection through endoscopic trans-sphenoidal approach is extremely crucial. Systemic anti-fungal drug should be taken immediately after diagnosis was ascertained.

## Patient consent

The patient has consented to the submission of the case report for submission to the journal.

## Abbreviations

MRI: magnetic resonance imaging
CSF: cerebrospinal fluid
ICA: internal carotid artery
NSAIDs: non-steroidal antiinflammatory drugs
